# Supplementary material for: Cerebellar microRNA-206 tunes Purkinje neuron firing dynamics to control sensorimotor gating
Source: bioRxiv. 2026 Jun 30:2026.06.29.734826. Preprint. [Version 1] doi: 10.64898/2026.06.29.734826 (PMC13345198; doi:10.64898/2026.06.29.734826)

bioRxiv preprint doi: https://doi.org/10.64898/2026.06.29.734826; this version posted June 30, 2026. The copyright holder for this preprint (which was not certified by peer review) is the author/funder, who has granted bioRxiv a license to display the preprint in perpetuity. It is made available under a CC-BY 4.0 International license.

SUPPLEMENTAL FIGURE LEGENDS

Figure S1. miR-206 expression in brain is undetectable outside of the cerebellum at baseline, after stress, or with NMDA receptor block. (A–D) *pre-miR-206* RNAscope signal is not found in coronal sections through medial prefrontal cortex (A), hippocampus (B), or ventral midbrain (C), but is only detected in the PC layer of cerebellum of adult male mice (D). Images were stitched from multiple tiled epifluorescence images. Scale bars: 1 mm. **(E–I)** Quantitative RT-PCR for mature miR-206 across brain regions in mice exposed to chronic restraint stress (E, F), forced swim stress (F), acute restraint stress (G), chronic social defeat stress (CSDS, susceptible and resilient groups), and subchronic administration of the non-competitive NMDA receptor antagonist dizocilpine (I). miR-206 was only detected in cerebellum across all treatment groups, and dizocilpine caused a slight reduction in expression in cerebellum. n = 6 control, 6 chronic restraint stressed male mice (E), 6 control, 6 chronic restraint stressed, 5 forced swim stressed male mice (F), 6 control, 6 acute restraint stressed mice (G), 6 control, 10 CSDS susceptible, and 11 CSDS resilient male mice (H), and 6 saline, 6 dizocilpine-treated male mice (I). *$p = 0.0207$, two-way ANOVA and Tukey's multiple comparisons test. mPFC, medial prefrontal cortex; NAc, nucleus accumbens; DS, dorsal striatum; Septum; Amyg., amygdala; Thal., thalamus; Hyp., hypothalamus; MB, ventral midbrain; Hip, hippocampus; PAG, periaqueductal grey; Residual, remaining brain tissue after other regions dissected; STN, subthalamic nucleus; Cx, cortex; Cb, cerebellum. Error bars indicate SEM.

Figure S2. miR-1 and *Calb1* expression are unaffected in miR-206 KO mice. (A–C) Quantitative RT-PCR detection of (A) *pre-miR-1-1*, (B) *pre-miR-1-2*, and (C) mature miR-1 in brain regions of WT

bioRxiv preprint doi: https://doi.org/10.64898/2026.06.29.734826; this version posted June 30, 2026. The copyright holder for this preprint (which was not certified by peer review) is the author/funder, who has granted bioRxiv a license to display the preprint in perpetuity. It is made available under a CC-BY 4.0 International license.

and miR-206 KO mice. mPFC, medial prefrontal cortex; AuCx, auditory cortex; NAc, nucleus accumbens; DS, dorsal striatum; GPe, globus pallidus external; Amyg, amygdala; Hyp, hypothalamus; Hip, hippocampus; vMB, ventral midbrain; Cb, cerebellum. n = 6 WT, 6 KO males. **(D–F)** *Calb1* RNAscope fluorescence intensity per PC (D) and averaged across all PCs per animal (E) are equivalent in WT and KO mice. n = greater than 2000 PCs per animal and 4 WT, 4 KO male mice. Representative tiled 10x epifluorescence images of sagittal cerebellar sections in (F). Scale bar: 500 μm.

Figure S3. Extended snRNA-seq and RNAscope data, related to Figure 3. (A) Feature plots of scaled expression of known cerebellar marker genes confirm Purkinje, astrocyte, granule, and oligodendrocyte (ODC) cluster identities. **(B)** Feature plots of scaled expression of markers of Aldoc (*Aldoc*) and Anti-Aldoc (*Rgs8*) PC subtypes. **(C)** Percentage of PCs assigned Aldoc and Anti-Aldoc identies in WT and miR-206 KO mice. n = 4 WT, 4 KO male mice. **(D–F)** RNAscope detection of *Aldoc* expression in WT and KO cerebellum. Representative tiled 10x epifluorescence images of sagittal cerebellar sections in (D). Fluorescence intensity per PC (E) and averaged across all PCs per animal (F) are equivalent in WT and KO mice. n = greater than 2000 PCs per animal and 4 WT, 4 KO male mice. Scale bar: 500 μm. **(G–H)** Volcano plots of differential gene expression in KO cerebellar nuclei (CN) (G), and all cells (H). Error bars indicate SEM.

Figure S4. Extended spatial-seq data and metabolomics, related to Figure 3. (A) Spatial feature plots of scaled transcript expression of astrocyte, Bergmann glia, molecular layer interneuron 1 (MLI), granule and oligodendrocyte (ODC) markers in representative WT (top) and miR-206 KO (bottom) cerebellar sections. **(B–E)** Volcano plots of differential gene expression in KO astrocytes (B), Bergmann glia (C), granule cells (D), and all cell types (E). **(F)** Spatial feature plot of scaled expression of *Slc17a6*, an excitatory cerebellar nuclei (CN) neuron marker, in WT and KO samples. **(G)** Volcano plot of differential gene expression in KO CN neurons (all subtypes) using C-SIDE analysis. **(H)** Gene-concept network plot showing fold change of genes differentially expressed in KO CN neurons connected with their respective enriched molecular function gene ontology pathways. **(I)** Heat plot showing fold change of genes differentially expressed in KO CN neurons mapped to enriched biological process gene ontology pathways. **(J)** Dot plot of Disease Gene Network (DisGeNET) terms enriched in genes that are differentially expressed in KO CN neurons. **(K)** Mass spectrometry of tricarboxylic acid (TCA) cycle intermediates shows elevation of lactate and succinate in KO cerebellum. (L–N): n = 12 WT, 10 KO male mice. *p = 0.0312 (lactate), **p = 0.0079

bioRxiv preprint doi: https://doi.org/10.64898/2026.06.29.734826; this version posted June 30, 2026. The copyright holder for this preprint (which was not certified by peer review) is the author/funder, who has granted bioRxiv a license to display the preprint in perpetuity. It is made available under a CC-BY 4.0 International license.

(succinate), multiple t tests + Bonferroni correction. **(L–M)** Mass spectrometry shows no change in neurotransmitter (M) or proteinogenic (N) amino acid levels in KO cerebellum. **(N)** Dot plot of DisGeNET terms enriched in differentially expressed genes for multiple cerebellar cell types. Error bars indicate SEM.

Figure S5. Male but not female miR-206 KO mice display stress-dependent hypolocomotion. (A–D) Male KO mice travel less distance in the open field chamber over 60 min (A–B) but vertical activity is unaffected (C–D). n = 32 WT, 28 KO mice. *p = 0.0192 (main effect of genotype), two-way RM ANOVA (A). *p = 0.0213, Welch's t-test (B). **(E–F)** Male KO mice travel less distance in a home cage across two night-day cycles. Grey: dark/active phase; yellow: light/inactive phase. n = 13 WT, 12 KO mice. ***p = 0.0002 (main effect of genotype), two-way RM ANOVA (E). ****p < 0.0001, two-way ANOVA (F). **(G–J)** Male KO mice travel less distance as compared to WT in the open field chamber following two seconds of acute restraint stress (G–H), but vertical activity is similar for WT and KO (I–J). n = 19 WT, 16 KO mice. ** p = 0.0030, two-way RM ANOVA (G). ** p = 0.0018, Welch's t-test (H). Data were analyzed for 60 min from time of restraint stress. **(K)** Body mass is equivalent in WT and KO males. n = 13 WT, 12 KO mice. **(L–O)** Female KO mice have no differences in baseline locomotion (L–M) or vertical activity (N–O) in the open field. n = 16 WT, 16 KO mice. **(P–Q)** Distance traveled in a home cage across two night-day cycles is similar for WT and KO females. n = 16 WT, 16 KO mice. **(R–U)** Distance traveled (R–S) and vertical activity (T–U) after acute restraint stress are equivalent in WT and KO females. n = 9 WT, 12 KO mice. **(V)** Body mass of females is not altered by miR-206 deletion. n = 16 WT, 16 KO mice. Error bars indicate SEM.

Figure S6. miR-206 deletion does not alter other stress- or anxiety-related behaviors. (A, D) Stress-induced hyperthermia is unchanged in miR-206 KO males (A) and females (D). n = 8 WT, 8 KO males; 7 WT, 9 KO females. ***p = 0.0002 (males), *p = 0.0111 (females) (main effect of time post- vs pre- stress; no main effect of genotype), two-way RM ANOVA. **(B–C and E–F)** miR-206 KO males (B, C) and females (E, F) are immobile in the tail suspension test for similar lengths of time as WT littermates. n = 16 WT, 10 KO males; 14 WT, 17 KO females. No effect of genotype by two-way RM ANOVA or Welch's t-test. **(G–J)** No effect of miR-206 deletion on duration in chamber (G), entry count H), distance traveled (I), or latency to enter light chamber (J) in a 10-minute dark-light emergence task. All mice spent less time, made fewer entries into, and traveled less distance in the light chamber. n = 19 WT, 16 KO male mice. ****p < 0.0001 (duration, entry count, and distance, main effect of chamber), two-way ANOVA. Latency was analyzed by Welch's t-test. **(K–N)** No effect

bioRxiv preprint doi: https://doi.org/10.64898/2026.06.29.734826; this version posted June 30, 2026. The copyright holder for this preprint (which was not certified by peer review) is the author/funder, who has granted bioRxiv a license to display the preprint in perpetuity. It is made available under a CC-BY 4.0 International license.

of miR-206 deletion on latency to eat (K), number of sucrose pellets consumed (L), number of fecal boli (M), or distance traveled (N) in a 30-minute hyponeophagia task. n = 16 WT, 14 KO males. Data analyzed by Welch's t-test. Error bars indicate SEM.

Figure S7. miR-206 KO male mice exhibit enhanced contextual fear memory retrieval. (A) Schematic of shock (US, unconditioned stimulus)- tone (CS, conditioned stimulus) pairings during fear conditioning, exposure to conditioning environment during contextual fear memory retrieval, and repeated 20-second tone presentations in a novel environment during cued fear memory retrieval. **(B, C)** Total percentage duration spent freezing during fear conditioning is unaltered in miR-206 KO males (B) and females (C). n = 23 WT, 25 KO males; 24 WT, 23 KO females (for all panels). **(D, H)** Freezing duration during fear conditioning rises similarly in miR-206 KO males (D) and females (H) as compared to WT following successive foot shocks. Gray bars indicate 30-second tone presentations with concurrent foot shock for the final 2 seconds. **(E, I)** Contextual fear memory retrieval is enhanced in miR-206 KO males (E) but not females (I). *p = 0.0422 (males, main effect of genotype). **(F, J)** Fear memory retrieval and extinction on a second day of exposure to contextual cues is not affected by miR-206 deletion in males or females. **(G, K)** Summary freezing duration across two days of contextual fear memory retrieval and extinction for males (G) and females (K). *p = 0.0495 (Day 1 WT vs KO males), Šidák's multiple comparisons test. **(L, O)** Cued fear memory retrieval is unaltered in miR-206 KO males and females on the first day of tone presentation. **(M, P)** Extinction of cued fear memory on the second day of tone presentation is delayed in miR-206 males but not females. *p = 0.0302 (genotype x time interaction), **p = 0.0056 (WT vs KO, 320 second time point), Šidák's multiple comparisons test. **(N, Q)** Summary freezing duration across two days of cued fear memory retrieval and extinction for males (N) and females (Q). All time-binned data were analyzed by two-way RM ANOVA. Summary data were analyzed by Welch's t-test or two-way ANOVA. Error bars and bands indicate SEM.

Figure S8. miR-206 deletion does not affect social preference in a three-chamber sociability test. (A) Male WT and miR-206 KO mice both spend a longer duration in a chamber containing an unfamiliar mouse than a chamber containing an empty basket. **p = 0.0058 (WT, mouse vs object), *p = 0.0283 (KO, mouse vs object). **(B)** Male WT and KO mice spend more time directly investigating the basket housing the novel mouse versus an empty basket. ***p = 0.0002 (WT, mouse vs basket), ***p = 0.0006 (KO, mouse vs basket). **(C)** Reduction in distance traveled by male KO mice did not reach significance (p = 0.0504), Welch's t-test. **(D)** Female WT and KO mice both spend more time in the chamber paired with a novel mouse versus an empty basket. *p = 0.0273 (WT, mouse vs

bioRxiv preprint doi: https://doi.org/10.64898/2026.06.29.734826; this version posted June 30, 2026. The copyright holder for this preprint (which was not certified by peer review) is the author/funder, who has granted bioRxiv a license to display the preprint in perpetuity. It is made available under a CC-BY 4.0 International license.

basket), ***p = 0.0003 (KO, mouse vs basket). **(E)** Female WT and KO mice both spend more time investigating the novel mouse-housing basket versus an empty basket. ***p = 0.0001 (WT, mouse vs basket), ****p < 0.0001 (KO, mouse vs basket). **(F)** Female WT and KO mice travel a similar distance during the test. n = 13 WT, 12 KO males; 16 WT, 16 KO females. All chamber and basket duration data were analyzed by two-way RM ANOVA followed by Holm-Šídák's multiple comparisons test. Error bars indicate SEM.

Figure S9. Extended sensorimotor gating and parvalbumin expression data, related to Figure 5. (A–C) Peak startle amplitude (A), latency to peak startle (B), and PPI (C) are unaltered in female mice with conditional deletion of miR-206 in parvalbumin-expressing neurons, including PCs and interneurons. n = 12 *miR-206*$^{fl/fl}$, 16 *miR-206*$^{fl/fl}$::*Pvalb*^{Cre} mice. **(D)** Parvalbumin (PV) immunofluorescence and DAPI staining in representative coronal sections of WT and miR-206 KO mice. **(E)** PV immunoreactive cell counts in medial prefrontal cortex, globus pallidus external, and dentate gyrus, CA1, CA2, and CA3 hippocampal regions of WT and KO mice. n = 4 WT, 4 KO male mice. **(F)** Quantitative RT-PCR detection of *Pvalb* mRNA in brain regions of WT and miR-206 KO mice (mPFC, medial prefrontal cortex; NAc, nucleus accumbens; DS, dorsal striatum; Septum; Amyg., amygdala; Thal., thalamus; Hyp., hypothalamus; vMB, ventral midbrain; Hip, hippocampus; Cb, cerebellum). n = 6 WT, 6 KO males. **(G–I)** Peak startle amplitude (G), latency to peak startle (H), and PPI (I) are unchanged in female mice with conditional deletion of miR-206 in PCs and retinal bipolar neurons. n = 9 *miR-206*$^{fl/fl}$, 15 *miR-206*$^{fl/fl}$::*Pcp2*^{Cre} female mice. Error bars indicate SEM.

Figure S10. miR-206 deletion does not alter levels of previously identified miR-206 target mRNAs in the RISC or translating ribosomes of Purkinje cells, or in other profiled brain regions. (A–C) Normalized sequencing counts of previously identified miR-206 targets for *Pvalb*^{Cre} (A) and *Pcp2*^{Cre} (B) HITS-CLIP and *Pcp2*^{Cre} TRAP-seq (C) experiments. n = 5 WT, 5 cKO mice (HITS-CLIP); 4 WT, 3 KO mice (TRAP). **(D)** Normalized TRAP-seq counts of targets previously identified by miR-206 sponge-mediated knockdown in PCs[28] are unaffected by conditional miR-206 deletion in PCs. n = 4 WT, 3 cKO mice. **(E–F)** Quantitative RT-PCR detection of *Estrogen receptor 1* (E) and *Brain-derived neurotrophic factor* (F) mRNA in brain regions of WT and miR-206 KO mice. mPFC, medial prefrontal cortex; AuCx, auditory cortex; NAc, nucleus accumbens; DS, dorsal striatum; GPe, globus pallidus external; Amyg, amygdala; Hyp, hypothalamus; Hip, hippocampus; vMB, ventral midbrain; Cb, cerebellum. n = 6 WT, 6 KO males. Error bars indicate SEM.

bioRxiv preprint doi: https://doi.org/10.64898/2026.06.29.734826; this version posted June 30, 2026. The copyright holder for this preprint (which was not certified by peer review) is the author/funder, who has granted bioRxiv a license to display the preprint in perpetuity. It is made available under a CC-BY 4.0 International license.

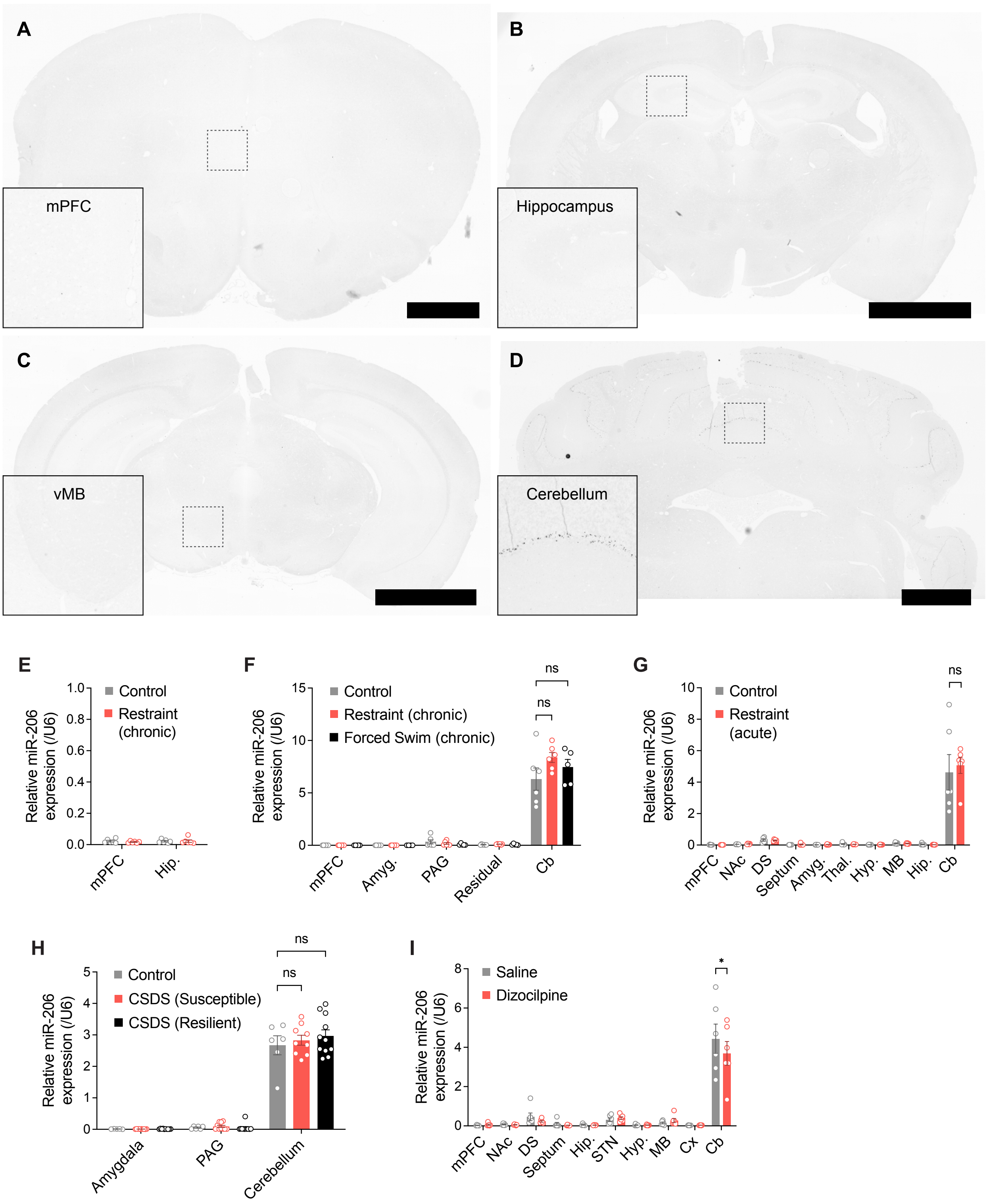

bioRxiv preprint doi: https://doi.org/10.64898/2026.06.29.734826; this version posted June 30, 2026. The copyright holder for this preprint (which was not certified by peer review) is the author/funder, who has granted bioRxiv a license to display the preprint in perpetuity. It is made available under a CC-BY 4.0 International license.

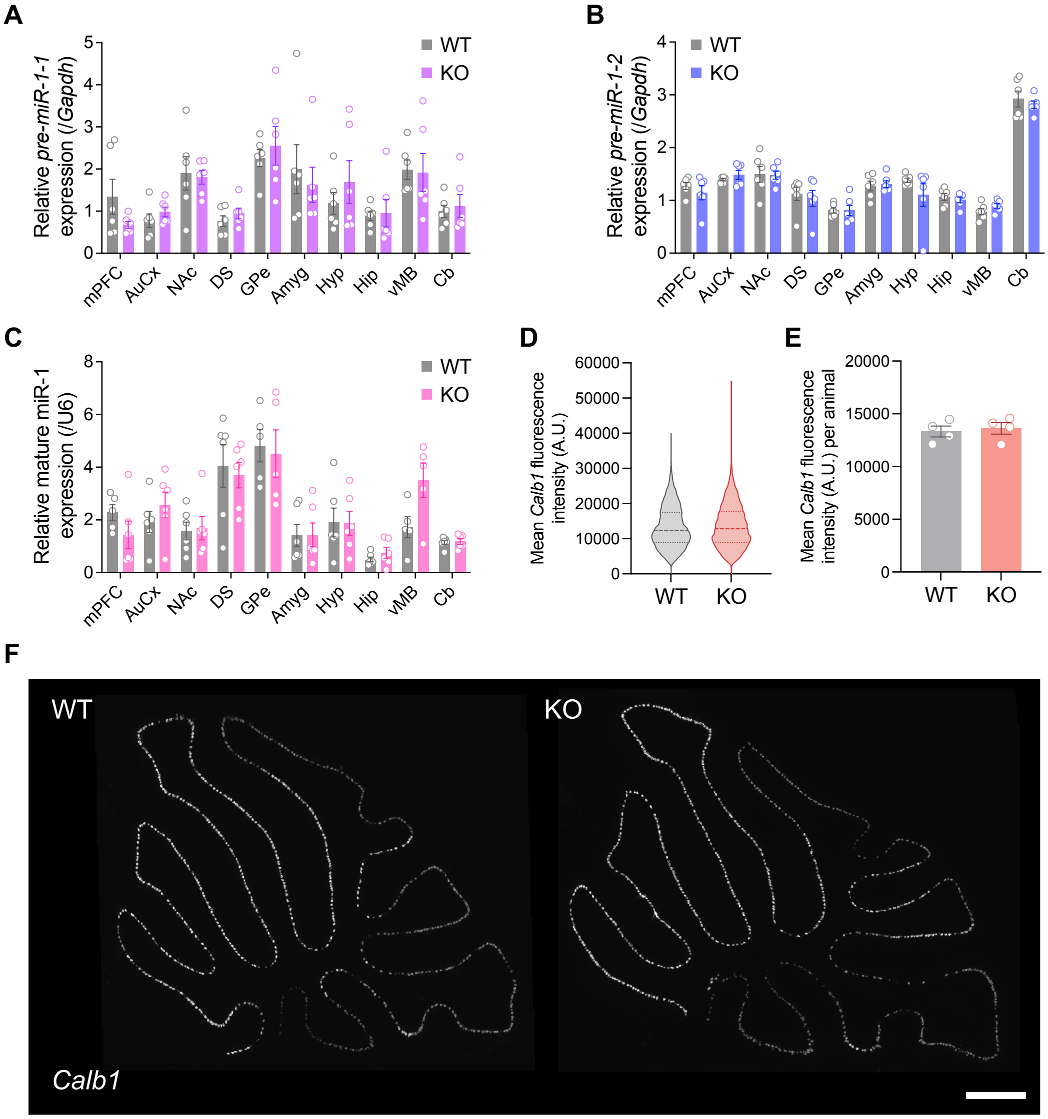

bioRxiv preprint doi: https://doi.org/10.64898/2026.06.29.734826; this version posted June 30, 2026. The copyright holder for this preprint (which was not certified by peer review) is the author/funder, who has granted bioRxiv a license to display the preprint in perpetuity. It is made available under a CC-BY 4.0 International license.

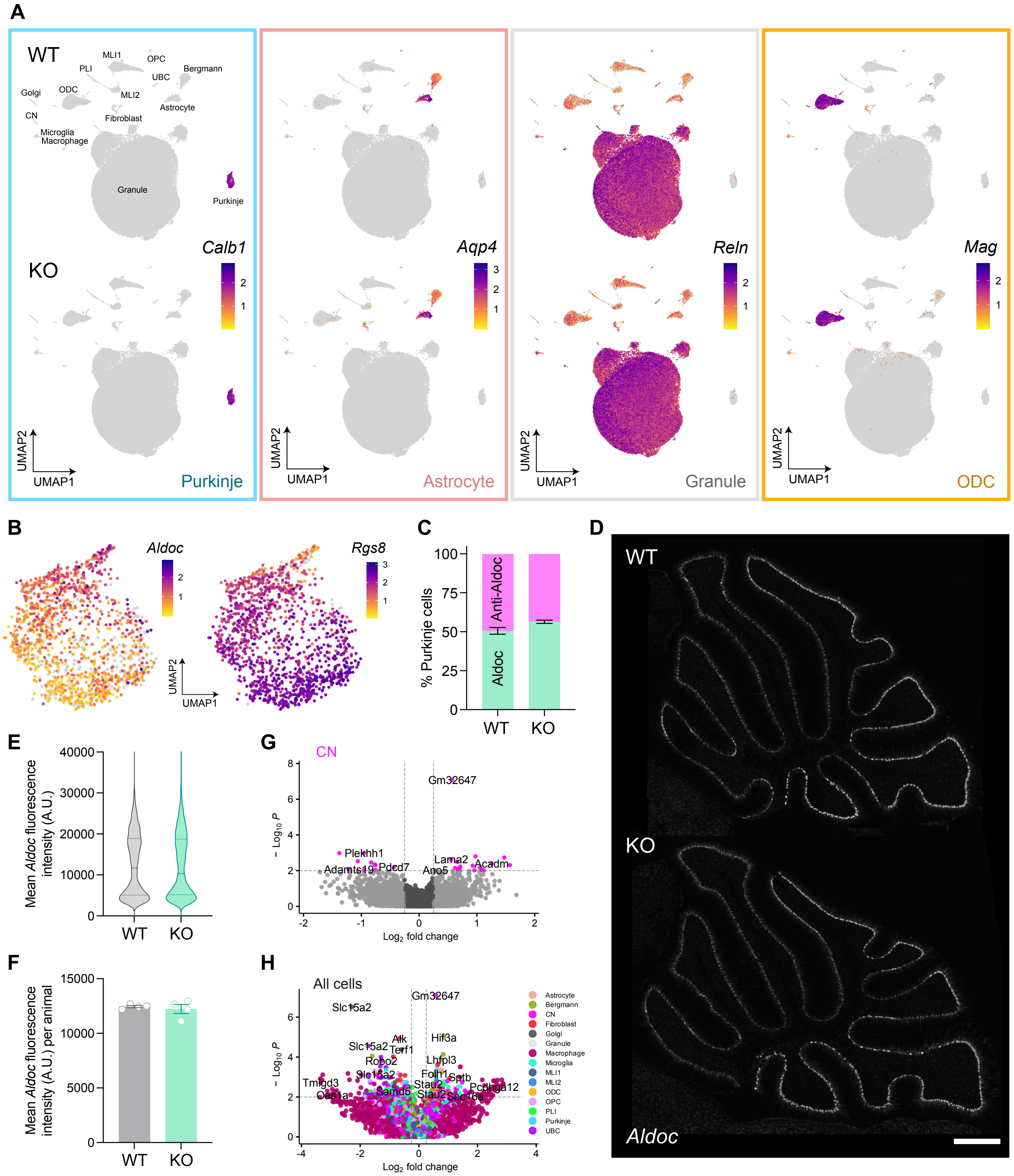

bioRxiv preprint doi: https://doi.org/10.64898/2026.06.29.734826; this version posted June 30, 2026. The copyright holder for this preprint (which was not certified by peer review) is the author/funder, who has granted bioRxiv a license to display the preprint in perpetuity. It is made available under a CC-BY 4.0 International license.

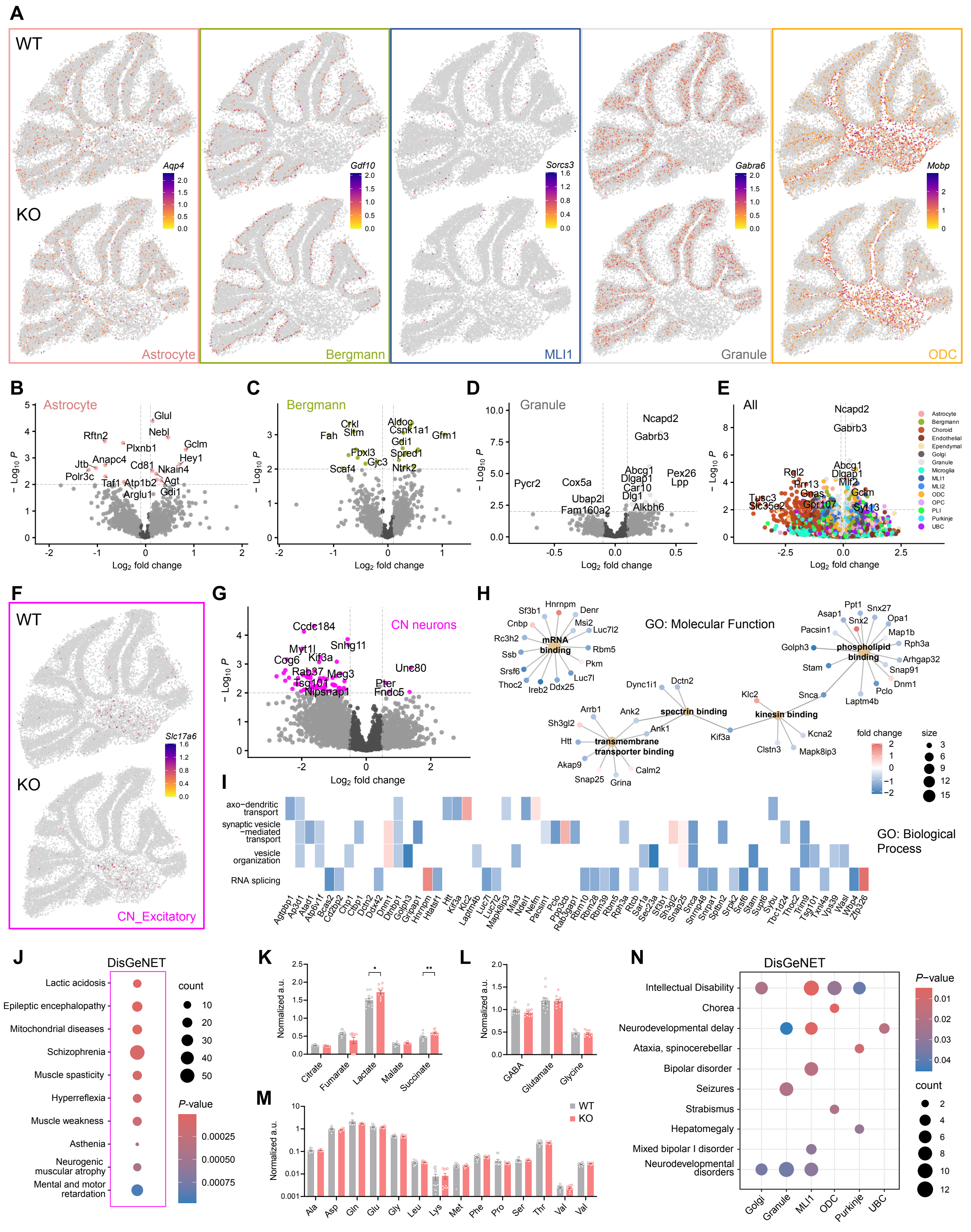

bioRxiv preprint doi: https://doi.org/10.64898/2026.06.29.734826; this version posted June 30, 2026. The copyright holder for this preprint (which was not certified by peer review) is the author/funder, who has granted bioRxiv a license to display the preprint in perpetuity. It is made available under a CC-BY 4.0 International license.

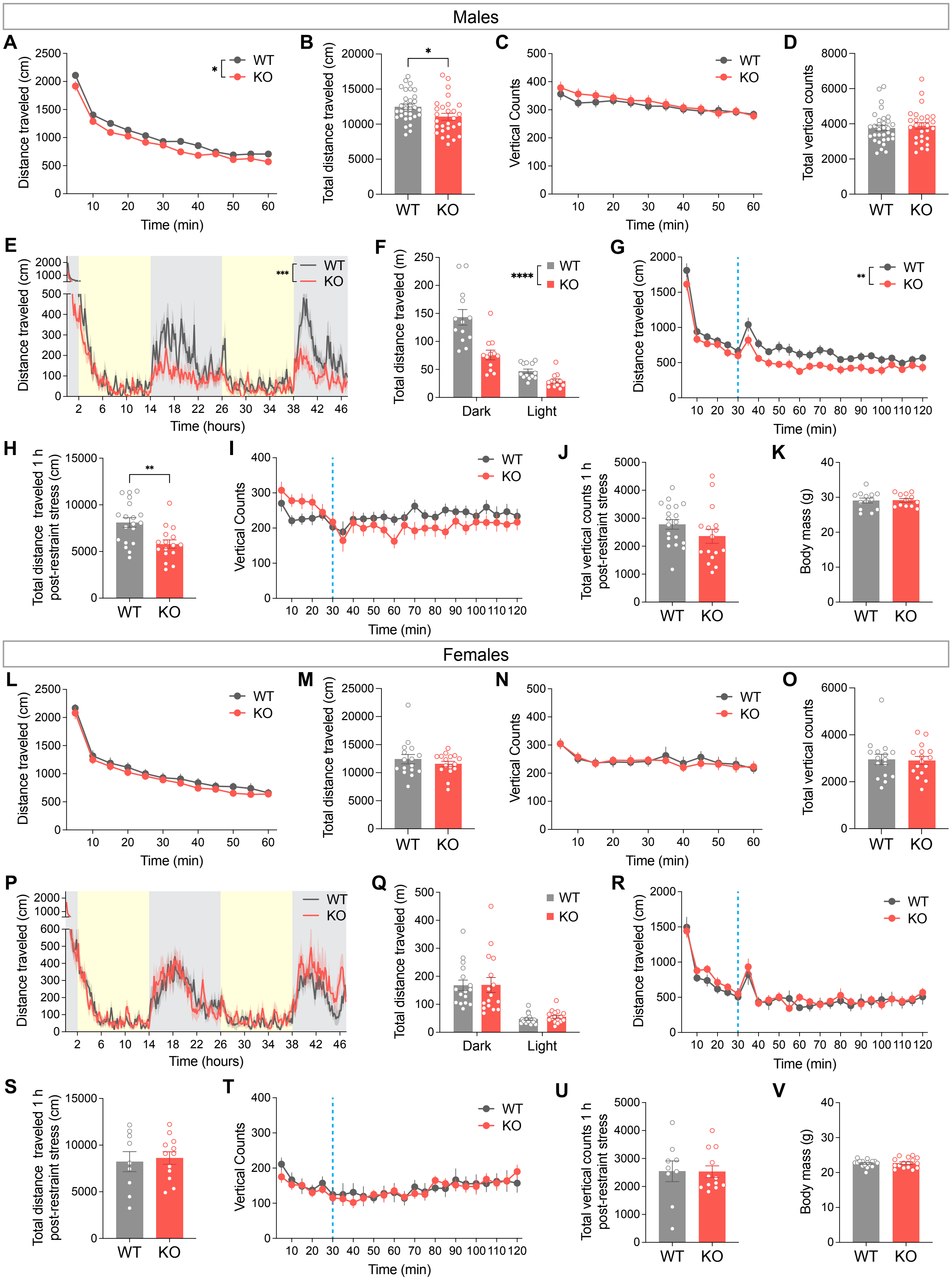

bioRxiv preprint doi: https://doi.org/10.64898/2026.06.29.734826; this version posted June 30, 2026. The copyright holder for this preprint (which was not certified by peer review) is the author/funder, who has granted bioRxiv a license to display the preprint in perpetuity. It is made available under a CC-BY 4.0 International license.

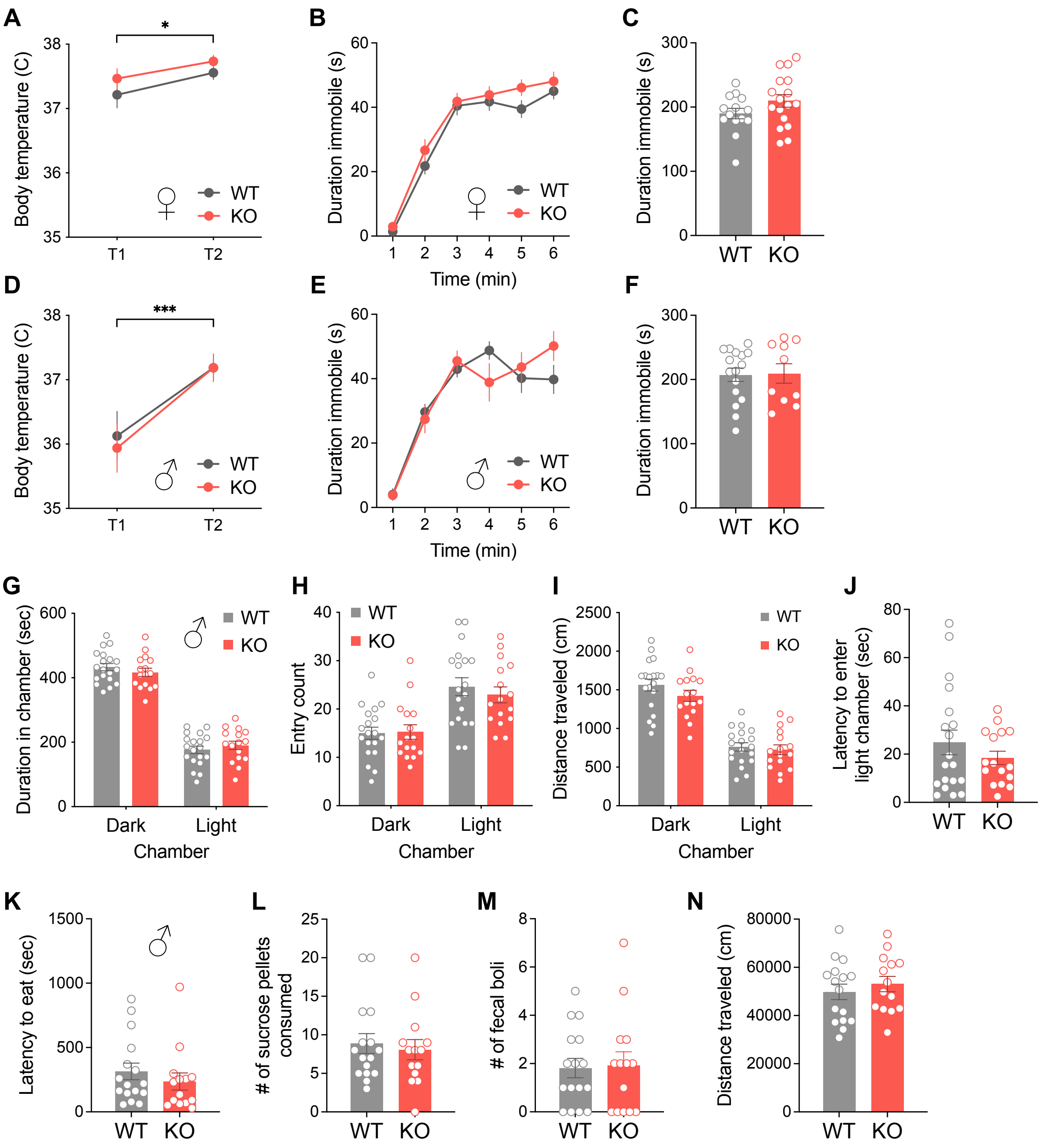

bioRxiv preprint doi: https://doi.org/10.64898/2026.06.29.734826; this version posted June 30, 2026. The copyright holder for this preprint (which was not certified by peer review) is the author/funder, who has granted bioRxiv a license to display the preprint in perpetuity. It is made available under a CC-BY 4.0 International license.

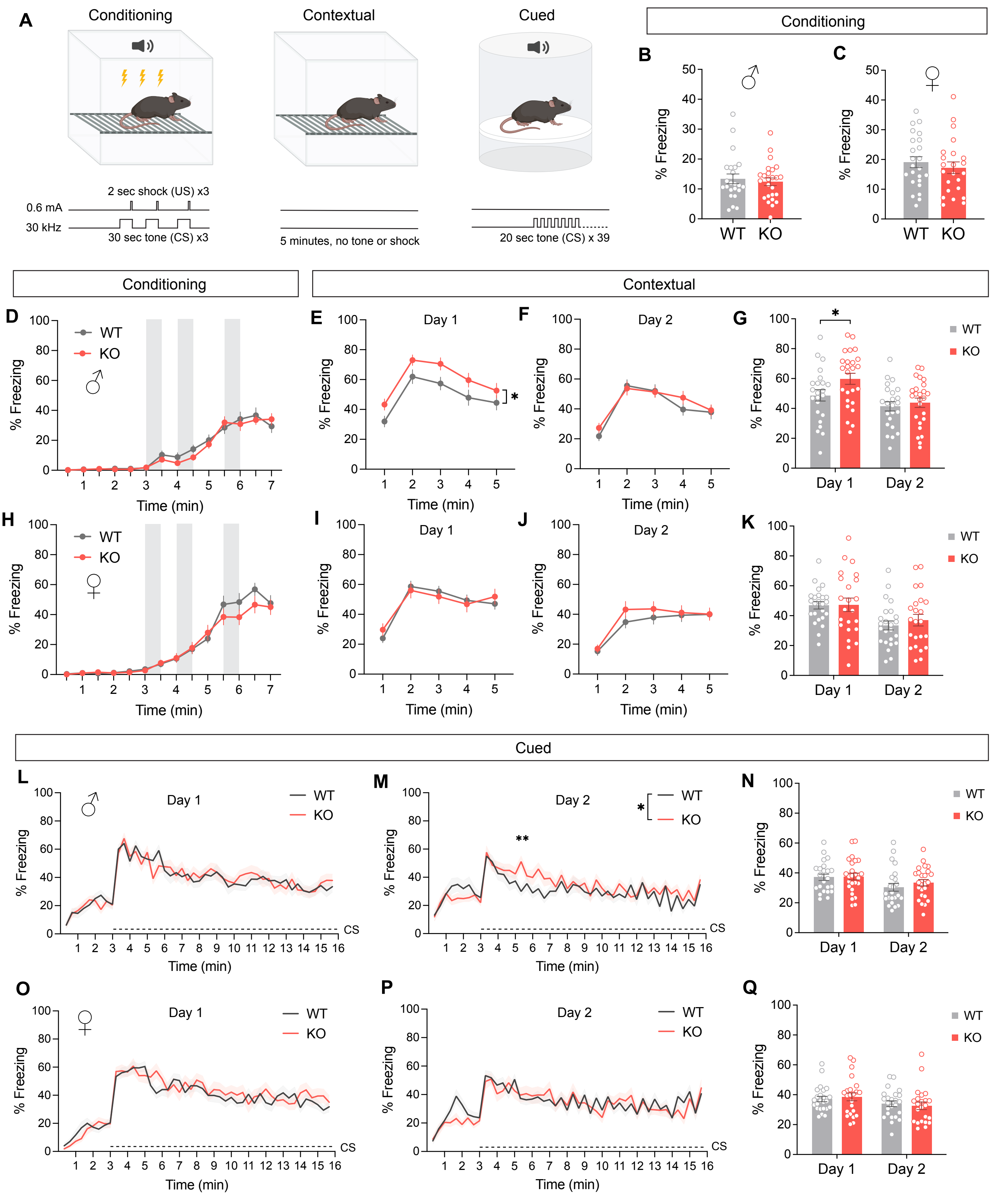

bioRxiv preprint doi: https://doi.org/10.64898/2026.06.29.734826; this version posted June 30, 2026. The copyright holder for this preprint (which was not certified by peer review) is the author/funder, who has granted bioRxiv a license to display the preprint in perpetuity. It is made available under a CC-BY 4.0 International license.

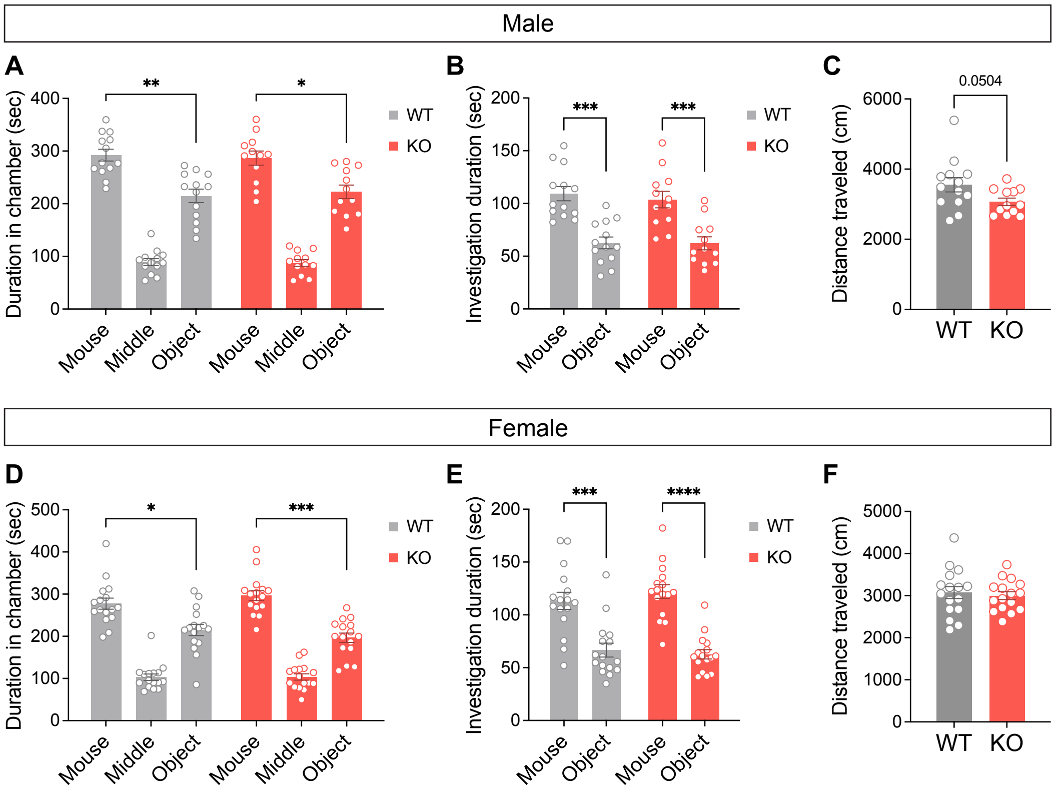

bioRxiv preprint doi: https://doi.org/10.64898/2026.06.29.734826; this version posted June 30, 2026. The copyright holder for this preprint (which was not certified by peer review) is the author/funder, who has granted bioRxiv a license to display the preprint in perpetuity. It is made available under a CC-BY 4.0 International license.

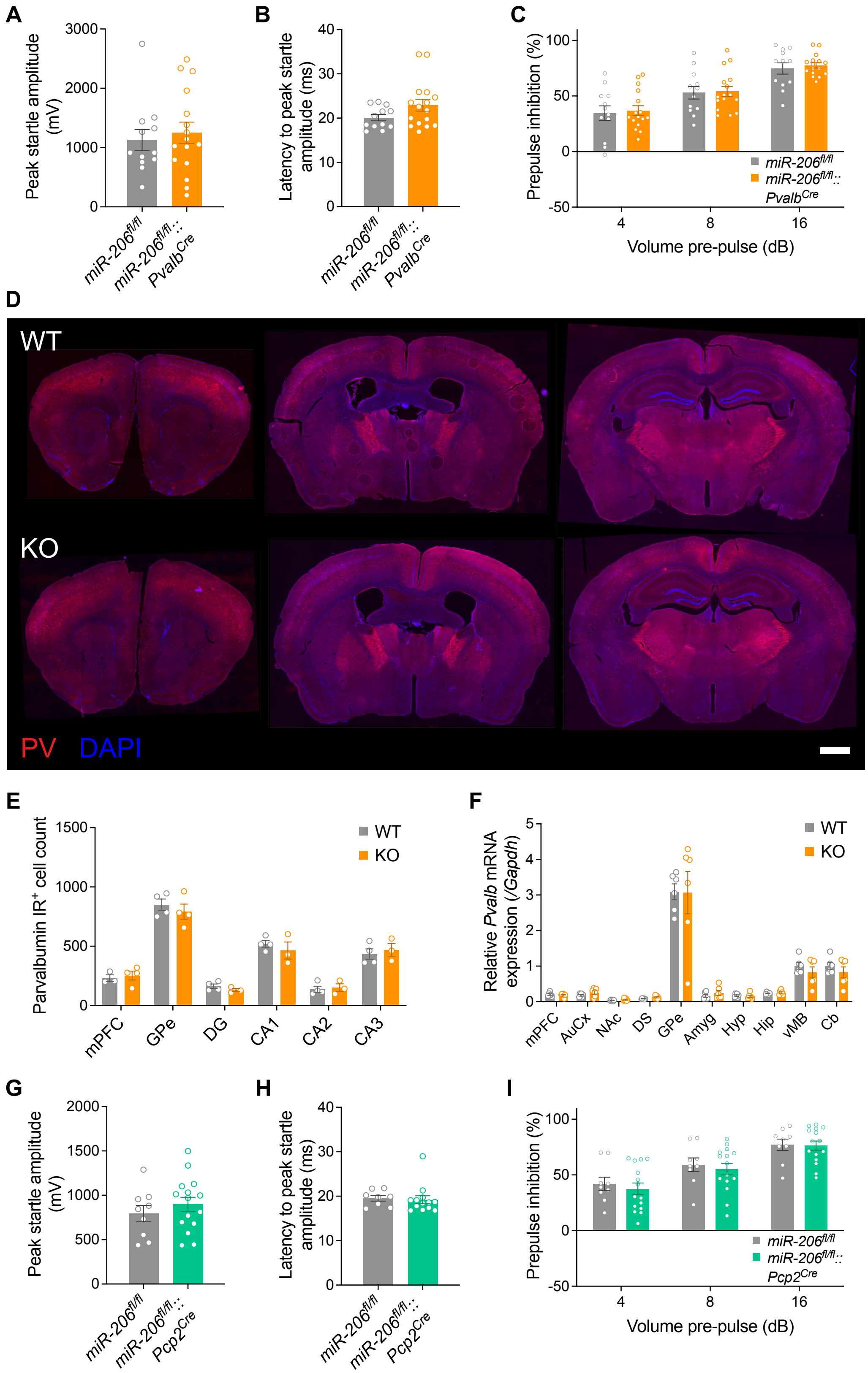

bioRxiv preprint doi: https://doi.org/10.64898/2026.06.29.734826; this version posted June 30, 2026. The copyright holder for this preprint (which was not certified by peer review) is the author/funder, who has granted bioRxiv a license to display the preprint in perpetuity. It is made available under a CC-BY 4.0 International license.

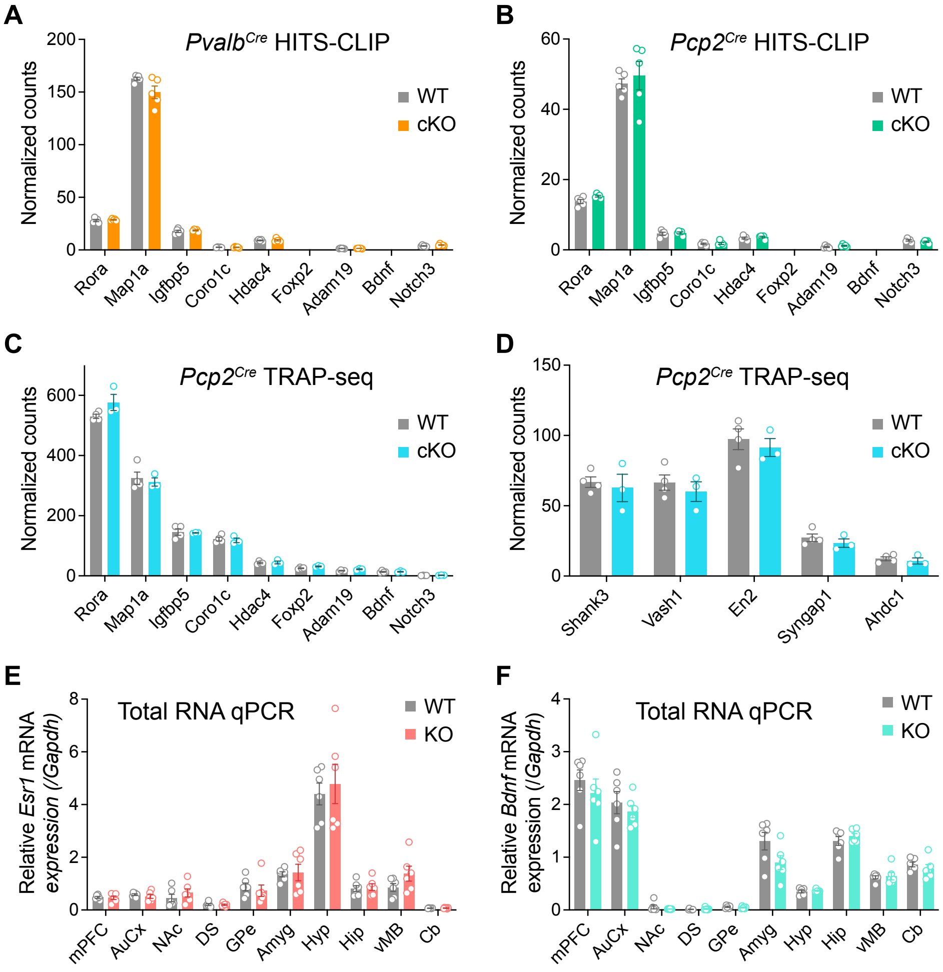

Supplement: Supplement 2 [file NIHPP2026.06.29.734826v1-supplement-2.pdf]
